# Spatial segregation of families with migrant background in the high-status City of Munich: How strong is the effect of socio-economic status?

**DOI:** 10.3389/fsoc.2023.1061975

**Published:** 2023-04-05

**Authors:** Michael Hanslmaier, Janna Teltemann, Michael Windzio

**Affiliations:** ^1^Referat für Stadtplanung und Bauordnung, Landeshauptstadt München, Munich, Germany; ^2^Institut für Sozialwissenschaften, Universität Hildesheim, Hildesheim, Germany; ^3^SOCIUM Forschungszentrum Ungleichheit und Sozialpolitik, Universität Bremen, Bremen, Germany

**Keywords:** segregation, integration, social distances, inequality, segregation indices

## Abstract

Ethnic residential segregation can result from preferential choices or from market forces. Depending on whether it evolved voluntarily or forcibly, segregation can have differential effects on immigrant integration. Using the example of Munich as a major German city, we examine the unequal spatial distribution of migrants and non-migrants. Following an approach proposed by Frank Kalter we calculate indices of residential segregation, which are adjusted for differences in socio-economic status. Results show that almost 14 percent of the residential segregation of immigrants can be attributed to socio-economic restrictions. This finding suggests that factors related to immigration and, possibly, also ethnic boundaries are determinants of the unequal spatial distribution also in high-status cities such as Munich.

## 1. Introduction

Assessing the causes of residential segregation empirically has long been difficult due to a lack of data and adequate methodological approaches. The common segregation indices do not allow controlling for group characteristics that might influence the unequal spatial distribution. For example, socio-economic status is an important factor for finding a place of residence, for immigrants as well as for non-immigrants. However, immigrants often have a lower socio-economic status than non-immigrants. A study that does not account for socio-economic status when analyzing ethnic residential segregation is likely to overestimate the impact of migration on residential segregation.

Using the example of Munich, a major German city, we examine the degree to which the dissimilarity index of spatial inequality of families with and without immigrant background is determined by differences in socio-economic status. The per capita purchasing power in Munich is highest in Germany. This wealth is a result of a very low unemployment rate of 4.5 percent and a steady increase of jobs (City of Munich/Landeshauptstadt München, 2022, p. 61). Munich's ethnically diverse population has grown from 1.36 million in 2006 to 1.56 million in 2016 and the growth is expected to continue in the future (City of Munich/Landeshauptstadt München, 2017a). Both developments put the housing market under high pressure (City of Munich/Landeshauptstadt München, 2016, p. 74). The question arises whether the processes of ethnic and socio-economic segregation follow a particular pattern in this rich city. For example, it might be that socio-economic status is a much stronger driver for segregation processes in Munich than in other cities. Also, immigrants who have on average a lower socio-economic status than non-immigrants might be at risk to live in stigmatized neighborhoods, which might have detrimental effects on their integration processes. In this paper, we aim at analyzing the risk of living in a stigmatized Munich neighborhood for persons with and without immigrant background. Further, we seek to decompose the Duncan index of segregation into a socio-demographic and a migration-related residual component.

Residential segregation of immigrants in cities has long been subject to sociological analyses. We contribute to this research by examining whether segregation of immigrants is the result of preferences, discrimination, or market forces. The understanding of the factors driving residential segregation can provide valuable insights into the degree of societal integration and can be used to develop measures to prevent undesirable trends from emerging. Only very few studies have been able to disentangle the determinants of segregation so far. In our paper, we explicitly shed light on residential processes in a high-status city (Munich).

The paper is structured as follows: we first give a brief overview of the concept of segregation and present theoretical accounts of segregation. After describing common segregation indices and ways of accounting for control variables, we refer to empirical findings on the extent and causes of ethnic segregation in Germany. This is followed by a description of our data base, the Munich Population Survey on Urban Development 2016. Based on factorial ecological analyses, we define stigmatized neighborhoods and analyze the risk of living in such a neighborhood by applying logistic regression. Secondly, we draw on a method proposed by Kalter (2001) in order to examine the extent to which ethnic segregation is reduced when socio-economic status characteristics are taken into account. We combine Kalter's approach with effect decomposition in logistic regression as described by Kohler et al. (2011). Our study concludes with a summary and critical discussion of our findings.

## 2. Theoretical accounts of segregation

The concept of residential segregation dates back to the early works of the Chicago School of Sociology in the 1920s and 1930s. Sociologists such as Robert Ezra Park and Ernest Burgess conceived the unequal distribution of ethnically homogeneous groups across the sub-areas of Chicago as a result of the competition for material and spatial resources (Burgess, 1968). Later studies pointed out that residential segregation is reinforced by mechanisms such as chain migration and networks (MacDonald and MacDonald, 1964). The emergence of ethnic infrastructures and labor markets further promotes the influx of immigrants (Collier, 2013). These mechanisms of residential segregation are often regarded as being “natural” or “preferential.”

Several studies provide evidence that only a small number of migrants considers geographical proximity to co-ethnics as a reason for choosing a place of residence (Nauck, 1988; Hanhörster and Mölder, 2000; Drever, 2004; Wiesemann, 2008). Rather, processes of spatial-social closure (“place stratification,” Alba and Logan, 1993) occur, for example, when private landlords discriminate against applicants with immigrant background, e. g. because they fear that land and building values could go down (Friedrichs and Triemer, 2008, p. 397). Moreover, migrant groups might respond to (anticipated) discrimination by “voluntarily” retracting into existing immigrant neighborhoods (Lamont and Mizrachi, 2012).

From an assimilationist perspective, residential segregation due to ethnic retention, preferences, or discrimination, is problematic since it indicates the presence of ethnic boundaries (Windzio and Trommer, 2018). In order to assess the extent and potential consequences of boundary-driven segregation, it is necessary to account for socio-economic disparities between locals and migrants. Socio-economic disparities cause residential segregation due to local differences in housing prices and rents. If immigrants have relatively fewer resources, they are often relegated to lower housing market segments (Farwick, 2012, p. 398). Empirically, socio-economic segregation is highly correlated with ethnic segregation.

## 3. The causes of ethnic segregation: Empirical findings from Germany

Friedrichs and Triemer (2008), as well as Friedrichs (2008) provide an overview of segregation in major German cities. Their analyses are based on the Duncan Index of Dissimilarity (Duncan and Duncan, 1955), which ranges from 0 to 1 (or 0 percent to 100 percent) and indicates the proportion of one group that would have to be redistributed in order to obtain an equal distribution (see below). In 2000 and 2005, Berlin, Dortmund and Dresden, among others, showed pronounced *ethnic* segregation with index values of around 0.30, whereas Bremen, Frankfurt, Munich and Stuttgart score rather low with index values of slightly above 0.10. The level of *social* segregation is generally lower in all cities, which suggests that there is also a migration-related component in the spatially unequal distribution. Comparisons between cities, however, are generally difficult if the number and size of administrative units differ.

Vom Berge et al. (2014) compared the segregation of low-income employees in 13 German cities with more than 500,000 inhabitants. Their analyses are based on grid cells, thereby allowing a better comparison between different cities. Results show that Munich exhibits the second lowest level of segregation (Duncan Index: 0.14) after Stuttgart. The highest levels are found for Frankfurt (0.20), Leipzig (0.19) and Berlin (0.18). The authors do not assess the extent to which social segregation is associated with ethnic segregation, although they point to the fact that districts with a high concentration of low-income employees often are also multiethnic districts.

Schönwälder and Söhn (2009) analyzed the concentration of different immigrant origin groups in 33 West German cities. They observed the highest concentration for Turkish foreigners. One third of this group live in urban areas in which at least 10 percent of the population are co-ethnics, while this is only the case for 5 percent of the Yugoslavs. However, the authors could not assess the causes of this concentration. Using logistic regression models, Janßen and Schroedter (2007) show that immigrants have a higher risk of living in a census district with a high ethnic concentration (more than 40 percent) than non-immigrants. The effect becomes stronger when socio-economic status is controlled. Sager (2011) examined the causes of spatial segregation of migrant groups with data from the German Socio-Economic Panel (supplemented by small-scale data). The results confirmed the concentration pattern of different immigrant groups as described by Schönwälder and Söhn (2009): concentration is highest for Turks and Italians. The findings also indicate considerable social segregation, since income and educational attainment of neighbors are correlated (Sager, 2011, p. 2625).

However, these small-scale individual-level studies cannot describe segregation at the level of cities, as the dissimilarity index does. Teltemann et al. (2015) analyzed residential segregation patterns in five German cities, among them Munich. In their individual-level data, neighborhoods have been identified by area codes. Among the cities of Dortmund, Kassel, Oldenburg and Stuttgart, Munich showed the second highest proportion of immigrants (36.1 percent), but rather low values of the Duncan Index. Following the approach of Kalter (2001, see below), the authors calculated a Duncan index, which was adjusted for socio-economic status. For Munich, the adjusted score was only about 13 percent lower than the unadjusted scores. Apparently, while being generally on a comparably low level, ethnic residential segregation in Munich is not mainly a result of socio-economic restrictions.

## 4. Segregation in a high-status city: The case of Munich

Munich is an economically prosperous city with a per capita purchasing power^1^ of almost 31.000 €, which is the highest value in Germany, ranking about one third above the national average. The local labor market experiences a steady increase of jobs. By 2030, Munich's population is predicted to exceed 1.8 million, corresponding to an increase of more than 16 percent of the value of 2015 (City of Munich/Landeshauptstadt München, 2017a). This growth takes place in a city that is already the most densely populated city in Germany (5,000 inhabitants/km^2^). Accordingly, the housing market in Munich is under high pressure. Rents have been rising from 10 €/m^2^ in 2005 over 16 €/m^2^ in 2015 to 21 €/m^2^ in 2020 (City of Munich/Landeshauptstadt München, 2022). Also the purchasing prices for real estate are rising sharply, leaving Munich the most expensive city when it comes to buying or renting in Germany. Furthermore, the increase of prices is accompanied by a decrease in the provision of social housing over the last 15 years (for a detailed analysis see City of Munich/Landeshauptstadt München, 2016). A similar development can be observed in most of the big cities in Germany, but at lower pace.

Despite the high overall wealth, a considerable proportion of the population in Munich is economically deprived. Depending on the definition, between 15 percent and 19 percent of the population in Munich can be categorized as being poor, i.e., they earn <60 percent of the average net equivalent income (City of Munich/Landeshauptstadt München, 2017c, p. 21, 23). Further, Munich is an ethnically diverse city (City of Munich/Landeshauptstadt München, 2017c, p. 25). In 2016, 28 percent of the Munich population did not have a German passport and another 15 percent were German citizens with migrant background. Munich's city administration, however, applies several measures to prevent ethnic and social residential segregation and to maintain the so called “Munich mix.” The social and ethnic structure of every district is intended to represent the city's overall distribution. In order to achieve this goal, building permits are subject to the requirement of providing 30 percent social housing. Further, the city maintains a portfolio of city-owned flats and promotes statutes to mitigate gentrification processes. This policy seems to be successful, as studies showed that the city's level of social segregation is relatively low (Vom Berge et al., 2014) and the level of ethnic segregation decreased between 1993 and 2005 (Friedrichs, 2008).

Given the high price level on the housing market and the political awareness for segregation, Munich is an interesting case to examine the causes of ethnic residential segregation and the living conditions of its residents. With regard to the price level, one could expect high ethnic segregation and a strong impact of socio-economic constraints on ethnic segregation. However, if political regulation supports social and ethnic mix, we might also find a lower impact of individual socio-economic conditions on housing decisions.

## 5. Methods: A decomposition of the Duncan-Index

Residential segregation leads to typical neighborhood compositions in which the concentration of groups with certain characteristics varies with the degree of segregation. Segregation and its effects are often discussed in terms of the consequences of living in a neighborhood with a high concentration of certain characteristics, such as immigrants or poorer individuals. Neighborhoods with these characteristics often develop a reputation or “stigma,” which may entail further effects for residents (e.g., discrimination based on address).

Before estimating the overall effect of migrant status on the segregation patterns in Munich we therefore first examine the probability to live in stigmatized neighborhoods for respondents with and without immigrant background. In a further step, we draw on the individual survey data and use a logistic regression model in order to estimate a “gross effect” of immigrant background on the risk of living in a stigmatized neighborhood. Subsequently, we include individual several indicators of socio-economic status into the model. See Section 6 and **Table 2** for a description of the variables used for the analyses.

If the effect of immigrant background changes to a significant extent after controlling for socio-economic and demographic conditions, we conclude that socio-economic status is a determinant of segregation and concentration processes. In order to be able to compare the non-linear regression coefficients, we computed average marginal effects (AME, cf. Mood, 2010; Best and Wolf, 2012).

This approach does not allow calculating the extent and determinants of segregation across the entire city. The second part of our analyses therefore focuses on the ethnic segregation pattern in Munich. Since the 1950s, various indices for measuring inequality in the distribution of groups over units (e.g., city districts) have been developed. With regard to residential segregation, different dimensions of unequal distributions across an urban area can be distinguished (Massey and Denton, 1988, p. 282ff.). A frequently used index is the dissimilarity index D (Duncan and Duncan, 1955) which offers a number of advantages compared to other measures. The calculation and interpretation is straightforward and corresponds to the concept of assimilation in terms of an “equal distribution” or “absence of differences” between groups (Kalter, 2001, p. 456; Kalter and Granato, 2004, p. 67).

D is defined as follows:


$$D=\frac{1}{2}\sum_{k=1}^{J}\left|\frac{A_{k}}{A}-\frac{B_{k}}{B}\right|$$

*J* indicates the number of categories (e.g., neighborhoods), *A* the number of persons of group *A* (e.g., natives), and *B* the number of persons of group *B* (e.g., migrants). *A*_*k*_ is the number of persons of group *A* in category *k, B*_*k*_ the number of persons of group *B* in *k*. D always takes values between 0 and 1. In the case of ethnic residential segregation, a value of 1 indicates that migrants and non-migrants never live together in a district. The index D can also be interpreted as the proportion of subjects (non-migrants or migrants) who would have to move in order to obtain an equal distribution across the districts (Duncan and Duncan, 1955, p. 211). Often, D is multiplied by 100 so that it represents the percentage of one of both groups that would have to relocate in order to achieve a balanced mix.

The value of D however depends on the number of spatial units and their size. Moreover, D is also influenced by the share of the respective group in the total population (Blasius, 1988). If the pattern of segregation does not correspond to the spatial units used for the calculation of the index, it is unreliable (Janßen, 2004, p. 20). Further, indices of segregation only represent descriptions of distributions (e.g., in a city) and, in contrast to regression methods, cannot account for confounding variables (such as socio-economic status) (Kalter, 2001). Kalter (2001) proposed a method addressing this shortcoming by combining multinomial logistic regression models (MNLM) with the Duncan index. However, this method requires individual-level data, whereas common estimations of segregation are based on aggregated data. In Kalter's approach, the column percentages required for calculating D are reproduced using the MNLM (Kalter, 2001, p. 459). Individual characteristics which might drive segregation can be accounted for by including them as independent variables in the MNLM. The dissimilarity index adjusted in this way is calculated as follows:


$$D=\frac{1}{2}\sum_{k=1}^{J}\left|\mathrm{Pr}(k|A,x)-\mathrm{Pr}(k|B,x)\right|$$

Here, Pr (k|A,**x**) and Pr (k|B,**x**) represent the conditional probabilities for migrants and non-migrants to live in a district *k*, which are a function of a vector of regression coefficients and a corresponding vector of covariates **x**. In our case, **x** also includes indicators of socio-economic status in order to capture the net effect of being a migrant or non-migrant. In other words, the interpretation of this adjusted dissimilarity index is conditional on the variables included in the model.

The calculated probabilities represent the column percentages (the proportion of each group living in a respective neighborhood) which are required for calculating the dissimilarity index D. Summed up over all neighborhoods, the probabilities amount to (approximately) 1, which is the prerequisite for calculating the dissimilarity index.

A similar procedure is proposed by Åslund and Nordström Skans (2008) taking the example of the unequal distribution of immigrants and natives across jobs in Sweden. Their starting point is the assumption that each workplace requires a certain individual realization of independent variables (e.g., education, language skills). Based on these characteristics, the authors estimated a propensity score which indicated the probability of being an immigrant holding a job with certain requirements. Subsequently, they generated a counterfactual distribution, which randomly assigned an immigrant status to individuals with a certain combination of characteristics. Based on this counterfactual distribution, it is possible to calculate different segregation measures and to compare them with the empirical measures.

Bayer et al. (2004) as well as Sager (2011) propose another method for calculating simulated measures of exposure, i.e., the probability of contact with members of one's own group in the neighborhood. In a first step, the proportion of immigrants in a person's own neighborhood has been estimated based on OLS regressions, where socio-economic characteristics have been controlled. In a next step, the regression coefficients have been used for predicting the proportion of immigrants for the average local population. The differences between the actual levels of exposure, adjusted for group differences in socio-economic status show the extent to which concentration is due to “ethnic” characteristics.

Another recent approach applies multilevel logistic regression in order to predict the probability of belonging to a particular group (Leckie et al., 2012; Spörlein and Schlüter, 2017). Model-based predictions with residential district-level random effects are used to simulate adjusted counts of group members in the respective neighborhood. In our view, the great advantage of this approach is the empirical Bayes shrinkage estimator of the multilevel model. In very small contexts where the reliability of the random effect is comparatively low, the estimated random effect is “shrunken” to the overall mean, so that contexts with more observations are assigned a higher weight than contexts with fewer observations. Thereby, the upward bias of D due to small cells in random samples is attenuated. However, if average marginal effects are used to calculate the decomposition (Teltemann et al., 2015), multilevel models compute the average of the random effect at u_0j_ = 0. Implicitly it assumes that the average individual lives in a district which is the average with regard to context characteristics—which is usually not the case.

The multilevel approach of Leckie et al. (2012) has the advantage of an implicit weighting by the number of observations per district, and it allows for assessing the impact of independent variables at different levels (e.g., neighborhood characteristics). Controlling for context-level determinants, however, is not relevant with regard to our research question. Neighborhood characteristics are the outcome of residential choices. We are interested in *individual* determinants of residential choices—e.g., ethnic group or income/education—thus, controlling for neighborhood characteristics would be endogenous. To give another example for this problem: assume an immigrant with low income moving into a particular neighborhood where rents are low. It is likely that low-rent neighborhoods show a higher concentration of less wealthy households and of immigrants. Yet the specific person's choice results from his or her income (and its relation to the low rent). If, in addition, we also accounted for the concentration of immigrants as a feature of this particular neighborhood, it would not be possible to determine the effect of economic constraints on segregation.

In order to identify the impact of socio-economic living conditions on segregation and to compute an adjusted index of segregation for the City of Munich, we therefore rely on the approach proposed by Kalter (2001). Our aim is to decompose segregation into a socio-economic and a residual, potentially migration-related component.

## 6. Data and analytical strategy

For our analyses, we draw on a representative survey conducted in Munich^2^ during autumn 2016. The survey is based on a random sample of 19.400 Munich residents aged 18 and older. Persons without German citizenship have been oversampled in order to compensate for the expected lower response rate of this group. A paper and pencil questionnaire was sent out by mail, but the respondents also had the opportunity to participate online.

To encourage participation of non-German speaking residents, the paper and pencil questionnaire has been translated into eight different languages (German, English, Polish, French, Italian, Croatian, Turkish and Greek) and the online version additionally offered a Russian and an Arabic questionnaire. Eventually, 5,945 respondents participated in the survey (response rate 31 percent). The translation of the questionnaire helped to boost the response rate of persons without a German citizenship compared to other studies. However, with about 18 percent, it is still below average. Nevertheless, due to the oversampling of the non-German population, the final sample contains 24 percent respondents without German citizenship, which comes close to the overall share in the population aged 18 years and older in Munich (see Table 1).

**Table 1:** Persons with immigrant background in survey sample and population of Munich.

	**Survey sample**		**Official statistics**
	**%**	**N**	**%**
Germans	67.3%	4,003	59.4%
German with immigrant background	9.1%	543	10.5%
Non-German citizens	23.5%	1,399	30.1%

Source: City of Munich/Landeshauptstadt München (2017b).

Population 18 years and older with main residence in December 2016.

In order to assess the impact of immigrant background on the probability of living in stigmatized neighborhoods, we have to define and identify neighborhoods and their stigmatization.

Therefore we follow the administrative definition of neighborhoods in Munich in the first step of our analysis (see **Table 4**). This definition distinguishes 475 small scale neighborhoods. The average population per neighborhood is 3,311 (minimum 2 and maximum 11,894). Excluding neighborhoods with missing values, our final individual-level sample consists of k = 360 neighborhoods with *N* = 4,822 persons. Stigmatized neighborhoods are commonly characterized by a high proportion of immigrants and high shares of households living below the poverty line (Teltemann et al., 2015). We compiled a list of neighborhood characteristics^3^ indicating stigmatization in different dimensions. This list comprises the proportion of persons with an immigrant background, as well as the proportion of children and young people under 18 with an immigrant background. In addition, we drew on several indicators of unemployment and poverty: the share of unemployed according to the German Social Insurance Code II and III among the 15–64 years-old population, as well among the age group 15–24. Moreover, we used the proportion of long-term unemployed persons of the unemployed population, the share of single parents in all households, and the share of persons in means tested poor households. Based on these indicators, we start our analyses with a factorial ecological analysis for 405 neighborhoods in Munich in order to differentiate between different dimensions of stigmatization.

Based on the factorial analyses, we define stigmatized neighborhoods as neighborhoods which belong to the fourth (highest) quartile of the distributions of the resulting factors.

After the identification of stigmatized neighborhoods we assess the probability of living in a stigmatized neighborhood for individuals with different characteristics, as well as the segregation pattern as described in Section 5.

The aim is to decompose the overall level of segregation into a “socio-economic” part and a “residual” part, which captures (among others) migration-related determinants. For this part of the analyses, we refer to a higher level of administrative units in our data (see the subsequent section), namely to Munich urban districts (*N* = 87). Drawing on this relatively large unit enables us to compare our analyses with results from previous studies (Friedrichs and Triemer, 2008; Teltemann et al., 2015). Further, we avoid small sample sizes by using districts instead of smaller neighborhoods, as we have to run separate regression models for every administrative unit. The average population^4^ per partial district is about 14,300 (minimum 108 and maximum 49,807). For the calculation of the unadjusted and adjusted dissimilarity index, we conduct a series of binary logistic regressions, one for each of the 87 districts. The respective dependent variable indicates whether a person is living (1) or not living (0) in a respective district.

In the first set of models, immigrant background is the only independent variable. In a second step, the indicators of socio-economic and demographic status are included (immigrant background, female gender, household income, squared household income, poverty, household size, squared household size, years living in Munich, living in partnership, years of education and academic degree). All control variables have been centered around their mean so that the intercept represents the *average* individual.

Finally, we are able to predict three (conditional) probabilities of living in the respective urban district:

1. The probability of an average person without immigrant background.
2. The probability of an average person with immigrant background *without* controlling for socio-economic and demographic status (“gross effect”).
3. The probability of an average person with immigrant background *after* controlling for socio-economic and demographic status (“net effect”).

**Figure 1:**
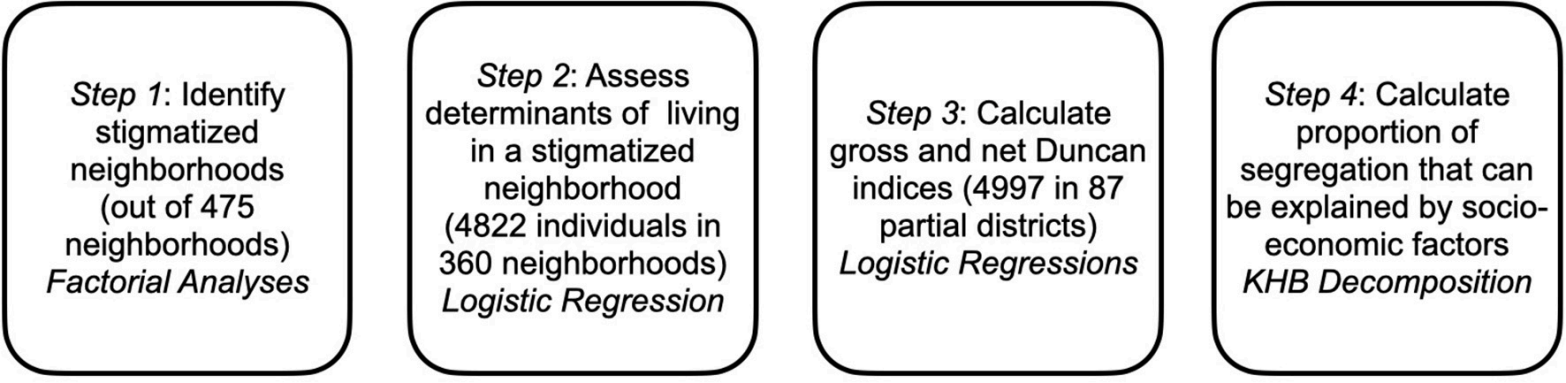
Steps of the analyses.

In addition to Kalter's (2001) approach, we calculate the differences between the gross and net effect of immigrant background according to the KHB (Karlson, Holm, Breen) method. This method, proposed by Kohler et al. (2011) is a simple procedure for decomposing effects in nested non-linear models, in order to ensure that coefficients of two different models are estimated with the same scaling. The full model [controlling for migration background (MB) and socio-economic status (SES)] is compared to a model in which the residual (R) of the regression of SES on MB is the predictor. The difference between R and SES lies solely in the component in SES which correlates with MB, and therefore R makes no independent contribution to the explained variance. So both models explain the same amount of variance of the underlying latent variable y^*^ (Kohler et al., 2011). Now, changes in β can be attributed to the confounders, whereas the rescaling does no longer affect the result. We used the KHB command in Stata 17 for calculating the adjusted segregation index. Figure 1 summarizes the steps of our analyses.

Table 2 gives an overview about the definition of the variables used for the analyses.

**Table 2:** Definition of variables, *N* = 4,822.

		**Mean**	**Std. dev**.	**Min**.	**Max**.
Stigmatized urban neighborhood (dv)	1 if both factor scores in Figure 2 are in the highest quartile,	0.17	0.37	0	1
Migrant background	1 if respondent -Non-German citizenship or -Immigrated after 1955, or -Has at least one parent who immigrated after 1955, 0 = Non-immigrants	0.38	0.49	0	1
Female	1 if Gender = Female, 0 = male	0.52	0.50	0	1
Age	Age in years	48.99	17.36	18	101
Household income/100	Household net equivalent income divided by 100	25.77	14.23	2	160
Household income sq./1,000	Squared household net equivalent income divided by 1,000	0.87	1.29	0.004	25.6
Poverty	1 if Household receives at least one of the following benefits: – General basic social care – Basic social care for job searchers – Unemployment benefits – Housing allowance	0.04	0.19	0	1
Owns two or more cars	1 if person owns more than two cars	0.21	0.40	0	1
Years of education	Educational degree in minimum required number of years	15.54	2.79	7	18
Academic degree	1 if person owns university or university of applied sciences degree	0.48	0.50	0	1
HHousehold size	Number of persons living in household including respondent	2.29	1.15	1	14
Years living in Munich	Number of years of Munich residency	27.39	21.37	1	94
Lives together with spouse	1 if respondent lives together with spouse	0.75	0.43	0	1
Female headed household	1 if head of household is single women	0.02	0.14	0	1
Context: multi-family house, 6–12 units	1 if respondent lives 6–12 units house	0.46	0.50	0	1
Context: multi-family house, 12+ units	1 if respondent lives 12 or more units house	0.34	0.47	0	1
Context: 1–2 family or house <6 units	Lives house with <6 units	Reference			

## 7. Results

The factorial ecological analysis based on seven indicators of deprivation for 405 neighborhoods resulted in two factors which we labeled as “diversity/immigration” and “poverty/unemployment” (see Table 3). Interestingly, after the oblique rotation both factors are considerably correlated (*r* = 0.37). One reason for this correlation is the fact that the proportions of single parents and long-term unemployed also load on the first factor “diversity/immigration.”

**Table 3:** Factorial ecology of immigration and poverty/unemployment, *N* = 405 small scale neighborhoods, oblique promax rotation.

**Variable**	**Diversity/ immigration**	**Poverty/ unemploym**.	**Uniqueness**
Percentage of persons with a migrant background as a percentage of the total population in 2016	0.8567		0.1857
Percentage of children and young people with a migrant background in the population under 18, 2016	0.8779		0.2018
Share of the unemployed SGB II + III in the population aged 15–64 years as a percentage 2016		0.8992	0.0589
Share of young unemployed (u25) SGB II + III in the population aged 15–24 in percent 2016		1.0103	0.0712
Proportion of long-term unemployed in the unemployed SGB II+III as a percentage 2015	0.5840		0.6865
Share of single parents in all households as a percentage 2016	0.6133		0.6629
Percentage of persons in means-tested poor households as a percentage of the total population 2016		0.7142	0.0827

Correlation of the two factors: r = 0.3755 blanks represent abs(loading) < 0.5.

**Figure 2:**
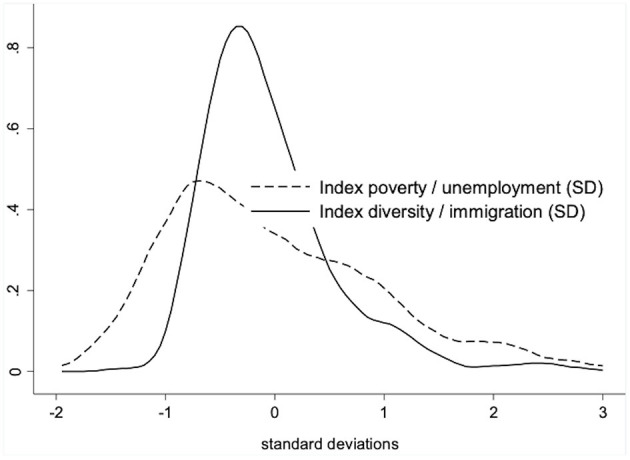
Distribution neighborhood factors.

Figure 2 shows the distributions of both factors. The distribution of diversity/immigration is slightly left-skewed and has a much smaller variance than the distribution of poverty/unemployment. In other words, while the immigrant population is distributed rather normally except for the higher concentration at the right tail of the distribution, poverty seems to be more evenly distributed, even though the right tail also indicates a concentration on some selected neighborhoods. The correlation of *r* = 0.37 indicates a tendency that some of these neighborhoods at the right tail of both distributions are actually identical across both distributions.

We define stigmatized neighborhoods as neighborhoods which belong to the fourth quartile of the distribution of the two factors. According to model 3 in Table 4 (see below), 16.8 percent of our sample live in stigmatized Munich neighborhoods. At the level of the 405 neighborhoods, 64 out 405 (15.8 percent) neighborhoods are stigmatized according to our definition.

**Table 4:** Determinants of living in a stigmatized urban neighborhood in Munich, logistic regression, average marginal effects, *k* = 360 small scale neighborhoods.

	**(Model 1)**	**(Model 2)**	**(Model 3)**
dv: living in stigmatized urban neighborhood			
Migration background	0.061^***^	0.038^***^	0.034^**^
Female	–	−0.022^*^	−0.019^+^
Age	–	−0.001^+^	−0.001
Household income/100	–	−0.006^***^	−0.006^***^
(Household income/100)^2^/1,000	–	0.037^***^	0.036^***^
Poverty	–	0.068^**^	0.063^**^
Owns two or more cars	–	−0.032^*^	−0.012
Years of education	–	−0.007^*^	−0.006^*^
Academic degree	–	−0.011	−0.011
Household size	–	0.000	0.007
Years living in Munich	–	0.001	0.001^*^
Lives together with spouse	–	0.024^+^	0.023^+^
Female headed household	–	0.068^*^	0.068^*^
Context: multi-family house, 6–12 units	–	–	0.063^***^
Context: multi-family house, 12+ units	–	–	0.121^***^
Context: 1–2 family or house <6 units	Reference	Reference	Reference
R2 (McKelveyandZavoina)	0.014	0.091	0.116
Observations	4,822	4,822	4,822

+ p < 0.1,

* p < 0.05,

** p < 0.01,

*** p < 0.001.

Who lives in a deprived urban neighborhood in Munich? Table 4 shows the results of three logistic regression models which estimate the odds of living in a stigmatized neighborhood. Model 1 in Table 4 only includes migration background as an explanatory variable, model 2 controls for socio-economic and demographic characteristics, whereas model 3 also includes the type of a person's residential unit. The coefficients are displayed as average marginal effects (Long, 1997) which indicate changes in the probability P(y=1|**x**), where *y* = 1 denotes living in a stigmatized neighborhood and x are the explanatory variables. The advantage of average marginal effects is that they allow the comparison of coefficients across different model specifications (Mood, 2010).

Without controlling for any confounders (Model 1), persons with an immigrant background have a 6.1 percentage points higher probability of living in a stigmatized urban neighborhood. Given that the overall share of stigmatized neighborhoods is 15.8 percent, this effect is noteworthy. The effect is considerably smaller in Model 2 where socio-economic and demographic characteristics are controlled: the higher the household income, the lower is the probability of living in a stigmatized neighborhood, but the negative effect tends to level off with higher values, as indicated by the positive effect of the squared household income^5^. Unsurprisingly, being poor (i.e., receiving welfare benefits) increases the probability of living in a stigmatized neighborhood. Similar to the effect of immigrant background, this result is not surprising as both characteristics contribute to the definition of the dependent variable. However, for our purpose of decomposing the determinants of residential segregation, it is interesting to see how the gross effect of immigrant background in model 1 changes after controlling for socio-economic and demographic characteristics in model 2. Moreover, also persons living in households with two or more cars, as well as persons with higher general education have significantly lower probabilities of living in a stigmatized neighborhood. While there is no additional effect of academic degrees, also female-headed households show a positive effect in model 3. Model 3 additionally controls for the type of residential units. Compared with the reference group of smaller houses with <6 units, living in a multi-family house with 6–12 units (0.063^***^) or in blocks with 12 and more units (0.121^***^) significantly increases the probability of living in a stigmatized neighborhood. Given the share of 15.8 percent stigmatized neighborhoods in Munich, particularly the latter effect is quite strong as the probability is increased by 12.1 percentage points. Obviously, the effects of the residential unit type describe the pattern of residential segregation with respect to the social inequalities in everyday living conditions. Stigmatized neighborhoods are more densely populated, people tend to live in larger buildings and are thus more exposed to their neighbors, e.g., in lifts and stairways, but are also more likely affected by noise or other negative externalities of their neighbors' everyday lives. Moreover, people in these neighborhoods have lower incomes and a lower level of education.

It is notable that the positive effect of migration background remains rather stable between models 2 and 3. However, a comparison between models 1 and 3 reveals that the average marginal effect decreased by 44.2 percent [(0.061–0.034)/0.061]. In other words, almost half of 1st and 2nd generation immigrants' residential location in stigmatized neighborhoods can be attributed to socio-economic and demographic characteristics.

Since the logistic regression estimates changes in *individual* probabilities conditional on **x**, it does not allow for a direct conclusion about patterns of residential segregation at the aggregate level. We address this issue in the last step of our empirical analysis. We estimate gross and net dissimilarity indices for Munich, based on 87 partial districts.

**Figure 3:**
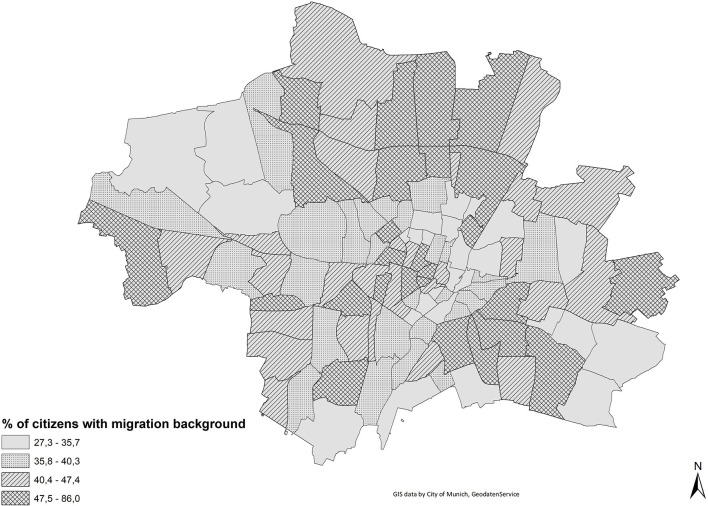
Proportion of migrants, grouped by quartiles.

First, we calculated the Duncan index of dissimilarity for ethnic residential segregation. Figure 3 shows the proportion of migrants (i.e., inhabitants without the German citizenship and Germans with migrant background^6^ combined) for every partial district, grouped by quartiles. The proportion of migrants differs between the north, the south east and the western part of the city center. The value of the Duncan index (based on administrative data) is moderate and amounts to 0.154.

**Figure 4:**
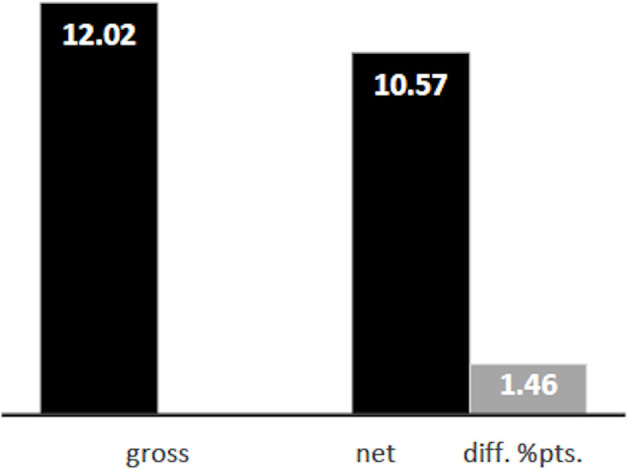
Gross and net level of segregation of persons with migration background, Duncan-Index.

Based on the survey data, we are able to reproduce the degree of residential segregation between persons with immigrant background and non-immigrants as measured by administrative data quite well: the Duncan index for the *gross* level of segregation of persons with migration background is D = 12.02 (Figure 4). 12.02 percent of the minority would have to be relocated in order to obtain an equal distribution across the urban districts. According to administrative data the corresponding Duncan index is 15.4. Differences between administrative statistics and our survey also result from the fact that our survey does not include respondents from every district and is also due to the fact that our database comprises only persons aged 18 or older.

Applying the KHB decomposition, we estimate a *net* level of residential segregation of D = 10.57, meaning that only 10.57 percent of the minority would have to be redistributed in order to obtain an equal distribution. This is a difference of 1.46 percentage points. The share of the Duncan index that can be explained by our set of explanatory variables, including income and education, is thus 13.8 percent:


$$\begin{array}{l} {\text{Duncan}}_{\text{Δ}}=\left((\text{gross/net})-1\right)*100\\ \;=\left((0.1202435/0.1056795)-1\right)*100=13.78 \end{array}$$

**Figure 5:**
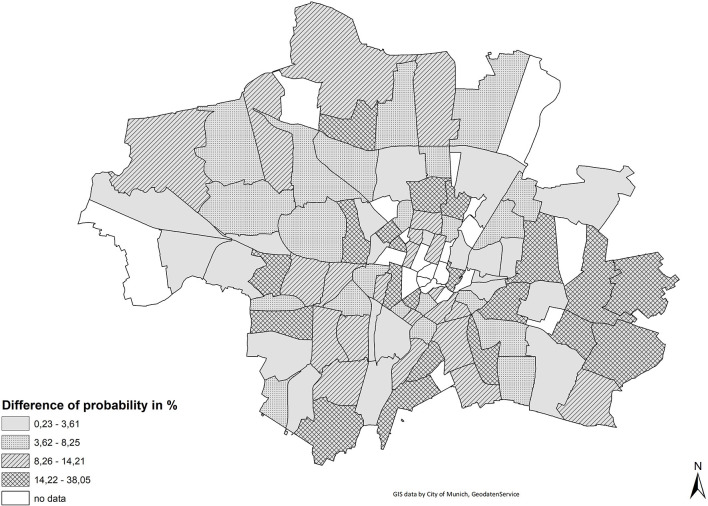
Probabilities of living in a district for a person with immigrant background when controlling for socio-economic status.

Figure 5 shows the difference of the probability of living in a respective partial district for a person with immigrant background when controlling for socio-economic status. A value of 15 percent means that the probability of living in that particular partial district changes by 15 percent adjusting for socio-economic and demographic status. This means that in areas with higher values a larger part of the residential distribution of immigrants can be attributed to socio-economic background characteristics. By contrast, partial districts where the probabilities do not change much are areas where immigrants are more likely to reside either due to their preferences or due to discrimination. Different mechanisms might explain the differences in the relevance of status, for example housing prices or housing preferences that are linked to household structure.

## 8. Summary and discussion

Compared with other German cities, the City of Munich is a special case. It is a high-status city with relatively wealthy inhabitants. It is ethnically diverse and benefits from a growing population. At the same time, rents in Munich are the highest in Germany. We analyzed living conditions of immigrants and non-immigrants and ethnic residential segregation in this outstanding spatial context. Our aim was to decompose the causes of residential segregation into a socio-economic and a migration-related component. While theories of immigrant integration explain why immigrants sometimes have a preference to live within neighborhoods mainly composed of their own ethnic group or of other immigrants, there might be also a strong socio-economic component in the segregation process, given the considerable socio-economic inequality between immigrants and natives. The comparison of average marginal effects in logistic regression models predicting the probability of living in what we have defined a “stigmatized neighborhood” showed that the effect of migration background decreased by 44 percent when controlling for a set of covariates related to income, poverty, education, and characteristics of the residential unit. However, these considerable changes in individual level probabilities are not reflected in the Duncan index when it is adjusted for socio-economic characteristics. We were able to demonstrate that 13.7 percent of the residential segregation of migrants is explained by socio-economic factors. This result is quite similar to findings of the study by Teltemann et al. (2015). Following from this, the socio-economic component in predicting residential segregation is not overwhelmingly strong, even if a high status city such as Munich is under study. As immigrants in Munich have a relatively high socio-economic status, market constraints might not affect them as much as in other cities.

In our study, we used Kalter's (2001) method of adjusting an aggregate measure such as the Duncan index according to individual characteristics. Recent developments in decomposing such measures come with the advantage that smaller residential units do not have to be dropped from the dataset just because they are small. Multilevel models for example take the variation of the reliability of the prediction due to different context sizes into account (Leckie et al., 2012). Further research should systematically compare the strengths and weaknesses of different methods of decomposition and quantify the benefit of each approach with regard to different data situations. For instance, one could argue that the multilevel approach allows controlling for neighborhood characteristics, such as the respective rent level. In our view, however, such an approach would be inappropriate regarding the aim of our study, namely the decomposition of economic effects from “residual” effects of immigrant status as a determinant of segregation. Actors decide on a residential location given their preferences. Certainly, their economic capacity often considerably limits their choice set. If we are interested in the question of whether levels of observed residential segregation do essentially result from socio-economic inequality, we must compute levels of segregation *net* of indicators of socio-economic status. According to our result, socio-economic inequality is a strong predictor of ethnic residential segregation in Munich, but is far from telling the whole story. The “residual” effects are much stronger and future research should focus on disentangling this residual, e.g., in terms of attitudes, values and other aspects of culture, as well as preferences regarding the educational infrastructure (Windzio et al., 2020). Moreover, in our analysis we applied the definitions of neighborhoods and districts as provided by administrative definitions. Recent studies provided a more sophisticated approach toward residential units. Future studies should elaborate how concepts of “fuzzy boundaries” (Legewie and Schaeffer, 2016) could contribute to the analysis of residential segregation.

## Data availability statement

The data analyzed in this study is subject to the following licenses/restrictions: data is restricted to use for employees of the City of Munich. Requests to access these datasets should be directed to plan.ha1-21@muenchen.de.

## Author contributions

All authors listed have made a substantial, direct, and intellectual contribution to the work and approved it for publication.

## Footnotes

^1^GfK study on purchasing power, http://www.gfk.com/insights/press-release/purchasing-power-germany-2018/, viewed 6th February 2018.

^2^The survey was conducted by the market research Company INFO GmbH on behalf of the City of Munich, Department of Urban Planning (see City of Munich/Landeshauptstadt München, 2017b).

^3^The neighborhood characteristics are derived from official statistics from the City of Munich (https://www.mstatistik-muenchen.de/stadtteilstudie/atlas.html). We considered only the 405 out of 475 neighborhoods with 400 and more residents to yield valid data for all interesting variables.

^4^Only persons with principal residence in the City of Munich in December 2016.

^5^The average household net equivalent income is 2,577 € and the range is between 200 to 16,000 (see Table 2). According to the results in Table 4 (but based on logit coefficients instead of average marginal effects), the flipping point of the effects of income and income-squared is at ~8,400 €. This means that households with this income have the lowest probability of living in a stigmatized neighborhood. Subsequently, this probability increases again with higher income. However, given the far-above average flipping point, the increasing probability with increasing income holds only for a very small and highly selective subpopulation.

^6^Germans with a migration background are defined as persons that hold the German citizenship and who migrated themselves, at least one of their parents migrated or who are children of bi-national parents.
